# A gender-emotion interaction multi-task network for depression recognition via transformer-based multimodal fusion

**DOI:** 10.3389/fpsyt.2026.1825268

**Published:** 2026-06-19

**Authors:** Yujuan Xing, Ruifang He, Xiaoli Cao, Ping Tan, Li Chen

**Affiliations:** 1 School of Digital Media (Computer), Lanzhou University of Arts and Science, Lanzhou, China; 2 Second Provincial People’s Hospital of Gansu, Northwest Minzu University, Lanzhou, China

**Keywords:** depression recognition, emotion valence, gender-emotion interaction, multimodal fusion, transformer attention

## Abstract

Depression is characterized by high prevalence, high recurrence, high disability and high mortality, which seriously affects people’s work and life. Among various behavioral biomarkers, speech-based features have gained increasing attention in depression detection due to their non-invasive nature, affordability, and rich capacity for conveying affective states. However, conventional depression recognition approaches rely solely on unimodal acoustic representations and largely overlook the influence of emotion and gender. To address this limitation, this study proposed a gender-emotion interaction multi-task network(G-EIMTNet) for depression recognition via transformer-based cross modal fusion. In the feature fusion stage, the deep representations of Mel-spectrograms were extracted using convolutional neural networks(CNN), and then the Maximum Correlation Minimum Redundancy (MRMR) algorithm was employed to select acoustic higher-order statistical features that were highly correlated with emotions and depressive states. These two types of features were then fused through the transformer attention mechanism. In the depression recognition stage, a depression recognition network for the interaction between gender and emotion was constructed based on a multi-task framework. Experiments on the AVEC2014 dataset showed that this approach outperformed the baseline model by 15.88% and 14.73% in accuracy and F1 score, respectively. Ablation experiments verify the effectiveness of multi-modal fusion and gender-emotion interaction.

## 1 Introduction

Depression is a prevalent affective disorder characterized by persistent sadness, fatigue, and feelings of hopelessness, often leading to diminished interest in work and daily activities. It also adversely affects sleep patterns and appetite, further contributing to impaired concentration, and in severe cases, it may result in suicidal behavior. According to the World Health Organization’s 2021 report, the COVID-19 pandemic has significantly impacted global mental health, with healthcare workers, frontline personnel, students, individuals living alone, and those with pre-existing mental health conditions being disproportionately affected (1). Studies have shown that there is a significant positive correlation between depression and the risk of death (2). Currently, the diagnosis of depression predominantly relies on patients’ subjective reports, psychiatric evaluations, and rating scales. These approaches are limited by low efficiency and strong subjectivity (3). The adoption of simple, efficient, and non-invasive automated depression recognition methods not only facilitates early intervention but also reduces post-onset resource allocation and losses. Furthermore, such approaches help protect patients’ privacy, alleviate their concerns, and increase treatment-seeking rates. Speech signals offer unique advantages including easy acquisition, non-contact, minimal invasiveness, device portability, and low usage restrictions. Additionally, speech contains rich emotional cues that can directly and conveniently convey affective states. Years of research have demonstrated the feasibility of identifying depression through the speech analysis.

In the current research on speech-based depression recognition, feature extraction is the key. Early studies mainly relied on hand-crafted acoustic features such as fundamental frequency (F0), energy, jitter and shimmer, which are prosodic features. These directly reflect the pathological manifestations of depressed patients, such as slower speech rate and flat intonation, and have clear physical meanings (4, 5). The study further confirmed that specific acoustic statistical indicators are of great significance for clinical interpretability in long speech segments (6). However, hand-crafted features often have high dimensionality and contain substantial redundant information; direct input to the model can easily lead to the curse of dimensionality. Therefore, feature selection algorithms such as maximum relevance and minimum redundancy (MRMR) are widely used in biomedical signal processing to remove irrelevant noise and retain the most discriminative biomarkers (7). With the development of deep learning, researchers have leveraged convolutional neural networks for processing Mel spectrograms, thereby effective capturing complex time-frequency features in speech (8). However, deep spectral features and hand-crafted features are heterogeneous information, and their fusion is a challenge. Simple concatenation ignores the temporal dependencies and nonlinear correlations among features. In contrast to simple concatenation, cross-modal attention and bidirectional interaction are introduced, which is more robust to small samples, long sequences and modal noise. Multi-modal Transformer fusion network (9) proved that Cross-Attention can effectively align heterogeneous modal information in speech emotion recognition task (10). applied this mechanism to depression detection, and achieved complementary enhancement between features through a text-guided cross-modal Transformer. These studies show that the attention mechanism can adaptively weight different feature streams and significantly enhance the recognition effect.

In addition, depression is associated with atypical processing of emotional stimuli, including perception, response, and memory encoding. It can also be conceptualized as a mood disorder characterized by negative attentional bias (11). Moreover, there are gender differences in depression, and women are at a higher risk of depression (12). Most of the current researches ignore the influence of gender and emotion on the depression representation, which affects the robustness of the model in complex scenarios (13). pointed out that gender bias can seriously weaken the fairness and accuracy of the depression recognition model (14). constructed a joint emotion-depression detection network and proved that emotion recognition task can help the model extract more robust features. Ali’s research results in multi-task learning (MTL) prove that joint modeling of emotional states can significantly improve the generalization ability of the model through shared representation (15).

Despite promising advances in deep representation learning, feature fusion, and multi-task learning for depression recognition, several limitations remain: (i) the adaptive fusion of heterogeneous features is still suboptimal, and (ii) the integration of gender and emotion-related information within a multi-task framework has been largely underexplored. Motivated by these gaps, this study proposes G-EIMTNet, a gender–emotion interaction-aware multi-task network designed specifically to overcome these two limitations. Specifically, a dual-branch feature extraction module is designed: one branch employs a convolutional neural network (CNN) to learn deep semantic representations from Mel-spectrograms; the other applies minimum redundancy maximum relevance (mRMR) feature selection to identify features strongly associated with depression. These two complementary feature streams are then dynamically fused via a transformer-based cross-modal attention module. Finally, a multi-task learning framework explicitly modeling the interplay between gender and emotion is incorporated to enhance depression recognition performance.

The main contributions of this paper are summarized as follows:

- A transformer-based cross-modal attention module is designed to effectively solve the problem that it is difficult to efficiently fuse the deep spectral features extracted by CNN and the hand-craft features selected by mRMR.
- G-EIMTNet is constructed, which significantly enhances the robustness and generalization ability of the model in complex scenes by mining the potential association between gender, emotion and depression.

The remainder of this paper is organized as follows. Related works for multimodal fusion of heterogeneous acoustic features and the influence of emotion and gender on depression recognition are given in Section 2. Section 3 introduces our proposed method. Experimental results and discussion are described in Sections 4. Conclusions are drawn in section 5.

## 2 Related work

### 2.1 Multimodal fusion of heterogeneous acoustic features

Multimodal fusion considers not only the interactivity across modalities but also the correlations among heterogeneous features within each modality. Current modal fusion strategies primarily fall into three categories: feature-level fusion (early fusion) (16), model-level fusion (mid-stage fusion) (17), and decision-level fusion (late fusion) (18). directly concatenated the deep spectral features extracted by CNN with the MFCC along the feature dimension. The experimental results on DAIC-WOZ and MODMA were significantly better than those obtained by using only MFCC or only deep spectral features (19). concatenated LPC and MFCC into a feature sequence and fed it into 1D-CNN and long short-term memory (LSTM) respectively to capture local spectral structure and long-term temporal dependencies. The study employed a simulated annealing metaheuristic search to learn optimal weights for each feature subset, thereby achieving weighted feature fusion. Experimental results demonstrate that, within the spectral domain, complementary information exists among the original spectrum, Mel-spectrogram, and MFCCs (20). The attention-based acoustic feature fusion network, named ABAFNet (21), jointly processed four complementary acoustic representations: the upper envelope, linear spectrogram features, Mel-spectrogram, and high-level statistical features (HSFs), feeding into CNN and LSTM. Late weighted fusion was performed via a weight adjustment module (WAM), enabling ABAFNet to outperform both single-feature baselines and simple concatenation on two clinical depression speech databases (22). used the attention mechanism to fuse the MFCC and the spectrogram at an early stage. The JTA framework, proposed by Li (23), fuses the log-Mel spectrogram with features extracted from a pre-trained model (WavLM) via transactive attention. The multimodal spatiotemporal attention framework proposed by Niu (24) demonstrated that jointly audio and video features, and explicitly capturing complementary information across modalities, yields superior depression severity prediction performance compared to unimodal baselines. Saji (25) proposed a hybrid framework that combines facial action units (AU) with interview texts, using the transformer architecture for multi-instance learning (MIL), and effectively leveraging the complementarity between visual features and text features through late fusion.

Collectively, these studies indicated that attention mechanisms have become a central focus in heterogeneous feature fusion research (26, 27). In depression speech analysis, particularly under data-limited conditions, attention-based fusion of deep spectral features and hand-crafted acoustic features demonstrates greater robustness than spectrum-only models (28). However, the existing fusion strategies are difficult to achieve fine-grained feature alignment between heterogeneous features. Especially when dealing with the significant differences in physical semantics and data dimensions between high-order statistical features and deep spectral features, traditional methods are unable to achieve deep semantic fusion. Therefore, this paper proposes a cross-fusion method based on transformer, which utilizes the self-attention mechanism to capture cross-modal heterogeneous correlations, thereby solving the problem of deep fusion.

### 2.2 Research on the influence of emotion and gender on depression recognition

Prior work has found that the speech of individuals with depression in different situations consistently exhibits the characteristics of emotional passivation such as loudness reduction, low fundamental frequency, slow speech speed and prolonged pause duration (29). The meta-analysis conducted by Dell’Acqua (30) provides the physiological underpinnings for emotional blunting. By validating the emotion context insensitivity (ECI) hypothesis, their study revealed a generalized reduction in the amplitude of the late positive potential (LPP) when depressed individuals were exposed to various emotional stimuli. These findings suggest that the behavioral coldness observed in patients is deeply rooted in a significant impairment of the brain’s capacity to process emotional information. Recently, there has been much research interest in enhancing the performance of depression recognition by analyzing emotion influence. Xing (31) proposed to combine mRMR feature selection and multi-task Bagging ensemble for depression recognition. It was found that some HSFs more reflected valence, while others were more stably related to depression. After the explicit introduction of emotion recognition task into depression recognition model, the stability and accuracy of depression recognition were improved. The MoE model proposed by Guo (32) extracted speaker related features and emotion related features respectively, and then migrates them to the depression corpus for fusion. On the self-built Chinese depression corpus and AVEC2014 corpus, MoE performs better than only using acoustic features. This research further indicated that conflating emotion-related features with speaker-specific features may obscure depression-relevant acoustic cues. Other studies also demonstrated that incorporating emotion-related task into depression detection can improve the accuracy and generalization performance of the model (33, 34).

The research of depression recognition revealed that acoustic feature differences, such as MFCC3, were statistically significant only in male participants, suggesting that depression’s acoustic manifestations are not fully consistent across genders (29). Yang systematically analysis gender and racial biases in machine learning models for mental health assessment based on speech behavior, finding substantial disparities in both acoustic feature distributions and self-reported symptom scores (e.g., PHQ-9) across gender groups in anxiety and depression detection tasks (35). A domain-adversarial training framework, proposed by Kim (36), treated gender as a nuisance domain and mitigated the depression detection model’s reliance on gender-specific cues via adversarial learning. Experiments on the E-DAIC demonstrated that this approach not only improved the overall F1 score by approximately 13% but also substantially narrowed the performance gap between male and female. Some studies, from the perspective of privacy protection, have reduced the influence of demographic information such as gender through speaker decoupling technology, in order to improve the performance of depression detection (37, 38). A recent review noted that in terms of indicators such as F0 and formants, female depressives often show a decrease in peak frequency and amplitude, while male depressives tend to exhibit an increase in amplitude variation and formant changes. When age and gender are explicitly controlled in modeling, the prediction accuracy of speech features for depression is often higher and more stable (39). Gender and emotions have a significant impact on the depression recognition. However, most current studies have conducted separate research on these two factors, without delving deeply into their correlation. Therefore, this paper proposes a gender-emotion interaction multi-task depression recognition framework, which enhances the model’s recognition performance by modeling the correlation between gender and emotions.

## 3 Method

### 3.1 Description of dataset

This paper adopts the AVEC2014 dataset, which consists of 300 task-oriented depression videos recorded in human-computer interaction scenarios, involving 84 participants (40). The AVEC2014 dataset is oriented towards two tasks: participants read an excerpt of the fable “The North Wind and the Sun” in German; and the participants answer one of a series of questions in German, such as: “What is your favorite dish?”, “What is your best gift and why?”, or discuss a sad childhood memory, etc. Each record in this dataset has a BDI-II depression score (ranging from 0 to 63) and arousal/valence/dominance scores. Arousal, valence and dominance are three dimensions that describe emotions. Valence evaluates an individual’s positive or negative emotional response to a specific object, person or situation. Arousal reflects the intensity of an individual’s emotional response, while dominance indicates the influence an individual has over their environment. Arousal, valence and dominance are annotated for each speech recording by 3–5 raters using FeelTrace (41), which are time-continuous and each frame or second of the recording was assigned an emotional score.

The main objective of this study is depression recognition, which is a binary classification. Therefore, we mark the records with a depression score higher than 14 as depressed and those with a score lower than 14 as normal. Early studies conducted by Valstar and Schuller revealed that valence exhibits the strongest correlation with emotional states, followed sequentially by arousal and dominance (40). Among these dimensions, valence is the most readily comprehensible. We calculated the average valence value [-1, 1] of time continuity for each audio recording with multiple raters’ annotations. If the score was greater than 0, the emotional valence label of recording was positive; otherwise, it was negative. We manually annotated gender labels based on the recordings. We enhance the speech data by adding noise to improve the robustness of the model.

### 3.2 Feature extraction

**Figure 1:**
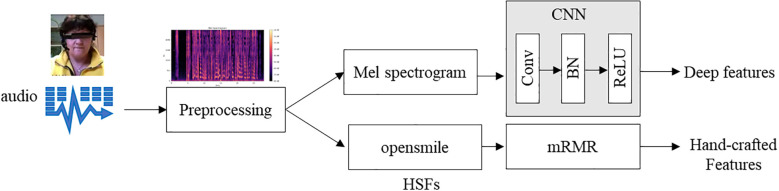
The procession of feature extraction.

The input features of the method proposed in this paper fall into two categories, namely, deep features and hand-crafted features. The feature extraction process is shown in **Figure 1**. In the stage of speech preprocessing, we employed a pre-emphasis function of $H(z)=1-\alpha z^{-1}$, which α=0.95. The overlaying framing approach was employed, with a Hamming window applied. The frame length and frame shift were set to 25ms and 10ms, respectively.

We obtained mel-spectrograms from audio data and scaled them to 256×128. We utilized a CNN to extract deep features, with the activation function being ReLU. For the extraction of hand-crafted features, this study employed OpenSmile and Librosa to derive low level descriptors(LLDs) from preprocessed audio data. These features included loudness, Mel-frequency cepstral coefficients (MFCCs), log Mel-frequency band power (logMelFreqBand), line spectral pair frequencies (lspFreq), fundamental frequency contour (F0finEnv), voicing probability of fundamental frequency candidates (voicingFinalUnclipped), fundamental frequency (F0), logarithmic energy(logEnergy), short-time energy(STE), zero-crossing rate(ZCR), formant center frequencies and bandwidths(F1/F2/F3/B1/B2/B3) along with their first- and second-order differences, as well as voice quality-related features such as jitter, shimmer, harmonic amplitudes and differences (A1, A2, A3, H1, H2, H4, H1-H2, H2-H4, H1-A1, H1-A2, H1-A3, HNR15), and cepstral peak prominence (CPP). High-level statistical function features (HSFs) were derived by applying functional statistics to these low-level descriptors (LLDs). The distribution of the LLDs and the corresponding HSFs adopted in this paper is shown in **Table 1**, where △ and △△ respectively represent the first-order and second-order differences of the relevant LLDs. **Table 2** lists the statistical functions used.

**Table 1:** Selected LLDs and their first-order and second-order differences.

LLDs	first-order and second-order differences
Spectrum related	MFCC(15)+△ lspFreq(8)+△ logMelFreqBand(8)+△
Energy related	Log energy(1) +△+△△ short-time energy(1)+△+△△ short-time zero crossing rate(1)+△+△△
F0 related	F0finEnv(1)+△ voicingFinalUnclipped(1)+△ F0final(1)+△ voiced sound (1) unvoiced sound (1)
Formant related	Formant center frequency (F1/F2/F3) (3)+△+△△ Formant center frequency bandwidth(B1/B2/B3) (3)+△+△△
Voice quality	jitterLocal(1)+△ jitterDDP(1)+△ shimmerLocal(1)+△ Harmonics to Noise Ratio (HNR) (1) loudness(1) +△ CPP (1) A1/A2/A3(3) H1/H2/H4(3) sound pressure level(SPL)(1) H1-H2/H2-H4/H1-A1/H1-A2/H1-A3(5)

**Table 2:** Statistical functions.

Category	Function
Statistic	max, min, mean, stddev, skewness, kurtosis, Max-Min, iqr2-3(quartile3-quartile2), quartile1,quartile2,quartile3,percentile1.0, percentile99.0, iqr1-3(quartile3-quartile1), pctlrange0-1, upleveltime75, upleveltime90, iqr1-2(quartile2-quartile1)
Regression	linregc1, linregc2, linregerrA, linregerrQ

Due to the high dimensionality of the extracted HSFs, we adopted mRMR (42) for feature selection. The core idea of the mRMR algorithm is to maximize the correlation between features and class labels while minimizing the correlation between features. That is say that, it selects features that are highly correlated with the class labels and have the least redundancy among themselves. We input the depression labels and valence labels into mRMR respectively to select features highly relevant to depression and emotion valence.

### 3.3 Proposed method

**Figure 2:**
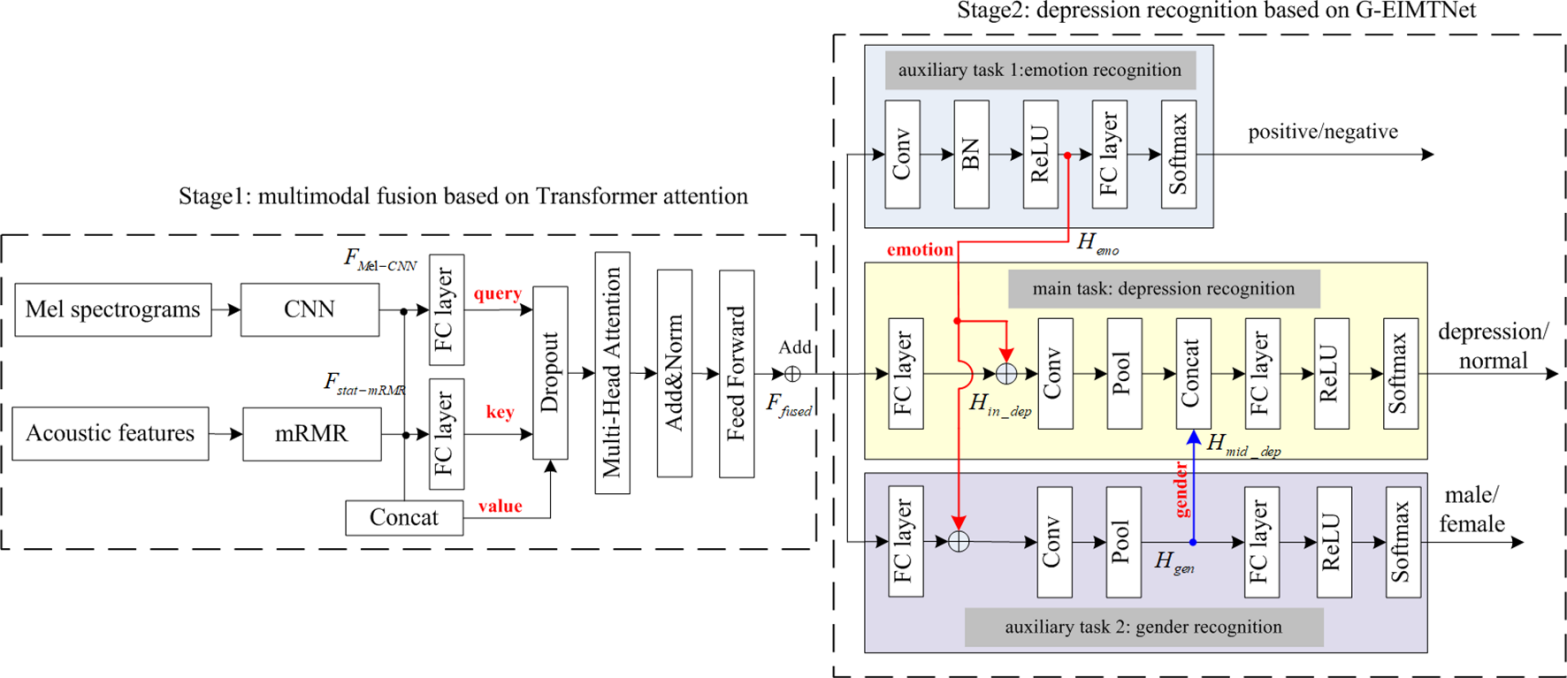
The multimodal depression recognition framework based on G-EIMTNet.

The proposed G-EIMTNet in this paper is mainly divided into two stages: multi-modal feature fusion and depression recognition. The framework is shown in **Figure 2**. Suppose that, the deep features extracted from the mel-spectrogram using CNN are denoted as $F_{\text{Mel}-\text{CNN}}$, the hand-crafted features are denoted as $F_{stat}$, and the features selected by the mRMR algorithm are denoted as $F_{\text{stat}-\text{mRMR}}$.

#### 3.3.1 Transformer-based multimodal fusion

The objective of this stage is to fuse deep spectral features with handcrafted acoustic features. In our transformer-based cross-modal fusion frame, Query (Q) is derived from $F_{\text{Mel}-\text{CNN}}$ and serves to search the focus. Key (K) is obtained from $F_{stat-mRMR}$, functioning as the indexing key. Value (V) is formed by the concatenation of $F_{\text{Mel}-\text{CNN}}$ and $F_{stat-mRMR}$, containing the complete original information. The calculations of Q, K and V are as shown in Equations 1–3:

(1) $$\text{ Query }(Q)=W_{q}\cdot F_{Mel-CNN}$$

(2) $$\text{Key }(K)=W_{k}\cdot F_{stat-mRMR}$$

(3) $$\text{Value }(V)=W_{v}\cdot \text{Concat}(F_{Mel-CNN},F_{stat-mRMR})$$

where $W_{q},W_{k},W_{v}$ denote the weight matrices for linear mappings.

Scaled Dot-Product Attention is used for computing attention weights, the formula is shown in Equation 4:

(4) $$\text{Attention}(Q,K,V)=\text{Softmax}(\frac{QK^{T}}{\sqrt{d_{k}}})V$$

where $d_{k}$ is the dimension of linear projection. In the feature fusion mechanism proposed in this paper, deep features are leveraged to query the importance of statistical features, followed by a weighting operation applied to the fused features. In fact, the Transformer uses multi-head attention. It does not perform the above calculation just once, but h times. The calculation is as shown in Equation 5.

(5) $$Attention_{multi-head}(Q,K,V)=Concat(head_{1},head_{2},\cdots ,head_{h})$$

where $head_{i}=Attention(QW_{i}^{Q},KW_{i}^{K},VW_{i}^{V}),\;\;(i=1,2,\cdots ,h)$. After the weighted addition, the features are processed sequentially through the Add&Norm and Feed Forward layers, yielding the final feature $F_{fused}$, which acts as the input to the subsequent stage. The formula is as shown in Equation 6:

(6) $$F_{fused}=Norm(Add(Attention_{multi-head}(Q,K,V)\cdot V))$$

#### 3.3.2 Gender-emotion interaction multi-task network

At this stage, emotion recognition and gender recognition are incorporated as auxiliary tasks to integrate emotion and gender information into the primary task (depression recognition), as illustrated in **Figure 2**. G-EIMTNet comprises three task branches, with information flow interaction facilitated via dedicated pathways.

In the auxiliary task of emotion recognition, the input feature $F_{fused}$ is fed into a convolutional network, which outputs the emotional valence (positive/negative) while extracting the feature map $H_{emo}$ subsequent to the ReLU layer. This feature map, which implies deep emotional information, is then transmitted to the gender recognition and depression recognition branches, as marked by red lines in **Figure 2**.

In the auxiliary task of gender recognition, the feature $F_{fused}$ is processed via a fully connected layer, followed by an element-wise addition with the emotion feature $H_{emo}$. The corresponding calculation formula is given as in Equation 7:

(7) $$H_{in\_gen}=\text{FC}(F_{fused})⊕\alpha H_{emo}$$

The purpose of doing this is to demonstrate the influence of emotional states on the acoustic manifestations of gender, such as anger or sadness which may mask the fundamental frequency characteristics of gender. In equation (7), α is a regulating parameter. While performing gender recognition, the feature map $H_{gen}$ after the Pool layer is extracted. This feature contains gender-specific acoustic patterns and, like $H_{emo}$, will be fed to the main task of depression recognition, as indicated by the blue line in **Figure 2**.

In the main depression recognition task, the fused feature $F_{fused}$ is first processed via a fully connected layer and then element-wise added to the emotional feature $H_{emo}$ to yield $H_{in\_dep}$. Subsequently, after passing through a convolutional layer and a pooling layer, the resulting feature is concatenated with the gender feature $H_{gen}$ to generate $H_{mid\_dep}$, which is ultimately fed into a fully connected layer for depression recognition (depression/normal). The calculation is as shown in Equations 8 and 9.

(8) $$H_{in\_dep}=\text{FC}(F_{fused})⊕\beta H_{emo}$$

(9) $$H_{mid\_dep}=\text{Concat}(\text{Pool}(\text{Conv}(H_{in\_dep})),H_{gen})$$

Prior to generating the final decision, G-EIMTNet explicitly leverages the gender feature $H_{gen}$ to calibrate features, thereby mitigating the issue of model gender bias. The training of the multi-task model is optimized using a joint loss function. The total loss $L_{total}$ consists of three parts, as shown in Equation 10:

(10) $$L_{total}=L_{dep}+\lambda_{1}L_{emo}+\lambda_{2}L_{gen}$$

where $\lambda_{1}$ and $\lambda_{2}$ are hyperparameters used to balance the contribution weights of the auxiliary tasks to the main task. $L_{dep}$, $L_{emo}$ and $L_{gen}$ denote the cross-entropy losses corresponding to the main task and the two auxiliary tasks, respectively. The calculation formula of cross-entropy loss is as shown in Equation 11:

(11) $$L=-\sum_{i=1}^{C}y_{i}\text{log}({\hat{y}}_{i})$$

#### 3.3.3 The training process of G-EIMTNet

By introducing the two stages of feature fusion and multi-task classification, the overall training process of G-EIMTNet was described as **Table 3**.

**Table 3:** The training process of G-EIMTNet.

Input	$X_{mel}$: Mel spectrograms $X_{acoustic}$: Acoustic features
Output	$D_{depression}$: Depression/Normal $D_{emotion}$: Positive/Negative $D_{gender}$: Male/Female
Process	# Stage 1: Multimodal Fusion based on Transformer Attention # 1. Feature extraction $F_{Mel-CNN}$=CNN_Extractor( $X_{mel}$) $F_{stat-mRMR}$*=* mRMR_Selector( $X_{acoustic}$) # 2. Mapped to the Transformer space Query = FC_Layer( $F_{Mel-CNN}$) Key = FC_Layer( $F_{stat-mRMR}$) Value = Concatenate( $F_{Mel-CNN}$, $F_{stat-mRMR}$) # 3. Feature fusion *X* = Dropout( Query, Key, Value) *X* = MultiHeadAttention(*X*) *X*= Add_and_Norm(*X*) *X* = FeedForward(*X*) $F_{fused}$*=* Add(*X*, Residual_Connection) # Stage 2: Depression Recognition based on G-EIMTNet # 1. Auxiliary Task 1: emotion recognition $H_{emo\_pre}$ = Conv_Block( $F_{fused}$) $H_{emo\_pre}$ = BatchNormalization( $H_{emo\_pre}$) $H_{emo}$ = ReLU( $H_{emo\_pre}$) $D_{emotion}$ = Softmax(FC_Layer( $H_{emo}$)) # 2. Auxiliary Task 2: gender recognition $H_{gen\_input}$ = FC_Layer( $F_{fused}$) # Feedback incorporating emotion information $H_{gen\_mid}$=Add( $H_{gen\_input}$, $H_{emo}$) $H_{gen\_mid}$ = Conv_Pool_Block( $H_{gen\_mid}$) $H_{gen}$ = $H_{gen\_mid}$ $D_{gender}$ = Softmax(ReLU(FC_Layer( $H_{gen}$))) # 3. Main Task: depression recognition $H_{dep\_input}$= FC_Layer( $F_{fused}$) # Integrate the emotional features from the auxiliary task $H_{emo}$ $H_{in\_dep}$=Add( $H_{dep\_input}$, $H_{emo}$) $H_{mid\_dep}$=Conv_Pool_Block( $H_{in\_dep}$) # Integrate the gender features from the auxiliary task $H_{gen}$ $H_{final\_dep}$ = Concatenate( $H_{mid\_dep}$, $H_{gen}$) # Final decision $D_{depression}$ = Softmax(ReLU(FC_Layer( $H_{final\_dep}$))) return $D_{depression}$, $D_{emotion}$, $\;D_{gender}$

Throughout the entire process, the CNN module used for processing Mel Spectrograms adopted a lightweight deep structure, consisting of 4 convolutional layers. Each layer was followed by Batch Normalization and ReLU activation functions. The first two layers used 3 ×3 convolution kernels, while the last two layers used 1 ×1 convolution kernels for channel reduction and feature integration. The input channel was 1, and the channel numbers of each layer were set to 32, 64, 128, and 256 respectively. After the 2nd and 4th layers, 2 ×2 max pooling was applied. In the transformer cross-attention module, the number of attention heads was 8, the input dimension was 256, the feedforward network dimension was 1024, and the dropout rate was set to 0.3. The training process used the AdamW optimizer, with an initial learning rate of $1\times 10^{-4}$. The batch size was set to 32, and the number of training iterations was set to 150.

## 4 Experimental results and discussion

In this section, we comprehensively tested the proposed depression recognition method, 5-Fold Cross-Validation was used for the experimental results, and accuracy and F1-score were selected as the performance evaluation metrics (43). Supposed that tp and tn represent the samples correctly predicted as positive and negative, respectively, fp and fn represent the samples incorrectly predicted as positive and negative, respectively. The formulas for calculating accuracy and F1-score are as shown in Equations 12 and 13:

(12) $$accuracy=\frac{tp+tn}{tp+fn+fp+tn}$$

(13) $$F1-score=\frac{2p*r}{p+r}$$

where $p=\frac{tp}{tp+fp}$, $r=\frac{tp}{tp+fn}$, represented the precision and recall of model. Accuracy metric quantified the ratio of accurate predictions generated by the model. F1-score integrated precision and recall, and a higher F1-score signifies the better classification performance.

### 4.1 1 Hand-crafted feature selection and analysis

In this subsection, the experiment analyzed 75 features highly correlated with depression and emotional valence obtained by mRMR, denoted as $F_{\text{stat}-\text{mRMR}}$. The feature distribution statistics were shown in **Table 4**.

**Table 4:** Feature distribution statistics of $F_{\text{stat}-\text{mRMR}}$.

Category	LLDs	$F_{\text{stat}-\text{mRMR}}$
Spectrum related	MFCC	30
	lspFreq	7
	logMelFreqBand	10
F0 related	F0	3
	F0final	2
	voicingFinalUnclipped	2
	F0finEnv	3
	voiced sound	1
	unvoiced sound	1
Energy related	short-time energy	5
	short-time zero crossing rate	2
Formant related	b3	1
	f2	1
Voice quality related	shimmer	2
	jitter	3
	H1, H1A1c	2

As shown in **Table 4**, among the 75 features, those associated with spectral characteristics, including MFCC, logMelFreqBand, and lpsFreq, account for 62.7% of the selected features, indicating a strong correlation between spectral features and both emotional valence and depression-related information. Notably, features such as the skewness and kurtosis of the first-order difference of MFCC, as well as upleveltimeX, were also frequently selected. Depressed individuals often exhibit psychomotor retardation: their facial and oral muscles tend to be flaccid, vocal organs move slowly, and speech lacks rapid, forceful state transitions. This manifests as reduced variability in kurtosis and significant shifts in skewness values. UpleveltimeX quantifies the fraction of time spent on rapid articulation within a speech segment. In normal communication, healthy individuals’ speech is marked by frequent phoneme transitions and clear enunciation, leading to frequent, prolonged high-value segments in MFCC first-order differences and thus higher upleveltimeX values. By contrast, the monotonous speech of depressed patients results in lower upleveltimeX.

**Figure 3:**
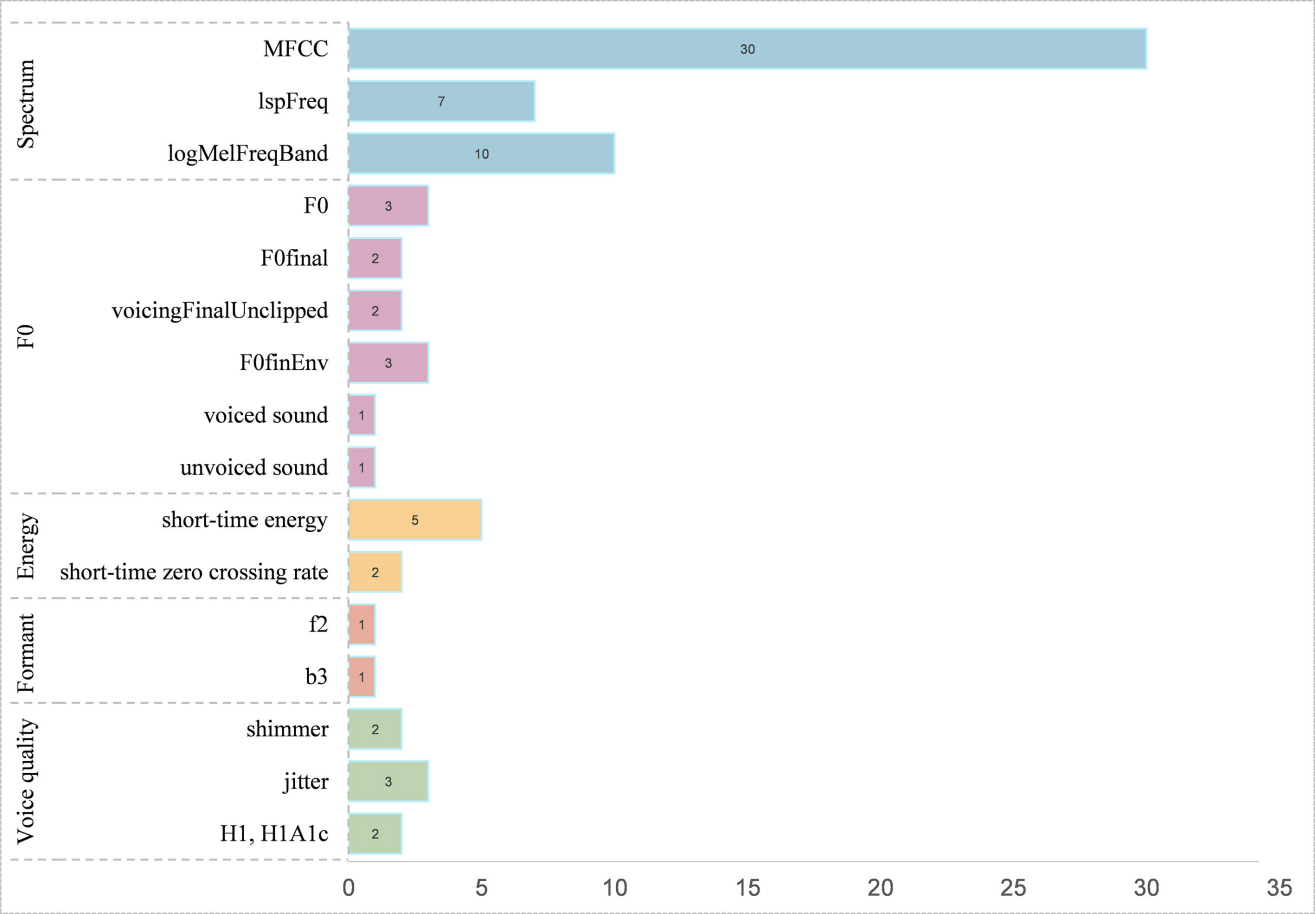
Select hand-crafted features.

Fundamental frequency (F0) and energy features capture weakened laryngeal muscle control and reduced lung capacity—consequences of emotional blunting and physical fatigue in depressed patients—directly reflecting the low-arousal state characterized by monotonous intonation (diminished F0 variability) and faint voice (energy attenuation). Voice quality features (e.g., Jitter, Shimmer, and H1) quantify vocal fold vibration instability and breathiness, which are canonical acoustic markers of negative emotional valence (e.g., anxiety, distress). In summary, this feature set accurately maps the abnormal states of depressed patients across emotional dimensions (valence and arousal) and physiological functions. The distribution of features is shown in **Figure 3**.

### 4.2 Ablation study for multimodal fusion

Ablation experiments were conducted to validate the effectiveness of transformer-based multimodal fusion. Specifically, four features were compared:

$F_{\text{Mel}-\text{CNN}}$: Only deep features extracted from mel-spectrograms were used;
$F_{\text{stat}-\text{mRMR}}$: Only handcrafted statistical features selected via MRMR were adopted;
$F_{\text{Hybrid}-\text{Concat}}$: Deep features and handcrafted features were simply concatenated;
$F_{fused}$: The Transformer-based multimodal fusion method proposed in this paper was employed.

All extracted features were fed into a fully connected layer followed by a softmax layer for classification. The experimental results are presented in **Table 5**.

**Table 5:** Comparison of different features.

Feature	Accuracy	F1-Score
$F_{\text{Mel}-\text{CNN}}$	0.7355	0.7129
$F_{\text{stat}-\text{mRMR}}$	0.7122	0.6851
$F_{\text{Hybrid}-\text{Concat}}$	0.7681	0.7453
$F_{fused}$	0.7950	0.7788

As shown in **Table 5**, for the single modality, $F_{\text{Mel}-\text{CNN}}$outperforms $F_{\text{stat}-\text{mRMR}}$, indicating that deep time-frequency features exhibit stronger representational capacity. $F_{\text{Hybrid}-\text{Concat}}$ outperforms both $F_{\text{Mel}-\text{CNN}}$ and Stat-MRMR, validating the complementarity between deep features and handcrafted features. The transformer fusion feature $F_{fused}$ achieved the best performance, with an accuracy of 79.5%, which is approximately 2.7% higher than that of simple concatenation. This indicates that the transformer attention mechanism has successfully established an effective mapping between heterogeneous features, which is conducive to enhancing the depressive expression of features.

### 4.3 Ablation study for G-EIMTNet

This study primarily evaluates the efficacy of incorporating emotional and gender information into depression recognition models. In the experiments, our comparative models include: a single-task depression recognition model (DEP_Indep), a multi-task learning model with only gender recognition as an auxiliary task (MTL-Gen), a multi-task learning model with only emotion recognition as an auxiliary task (MTL-Emo), and our proposed G-EIMTNet. The input feature is $F_{fused}$ and the experimental results are shown in **Table 6**. We fixed the main task training until convergence, then gradually introduced the emotion and gender branches, observing the changes in the F1 score of the validation set, and searching for the optimal balance point among the weights of each task. After multiple validation, the optimal values of $\lambda_{1}$and $\lambda_{2}$ in the total loss $L_{total}$ of G-EIMTNet are 0.65 and 0.35 respectively.

**Table 6:** Ablation study for G-EIMTNet.

Model	Accuracy	F1-Score
DEP-Indep	0.7750	0.7581
MTL-Gen	0.7924	0.7854
MTL-Emo	0.7953	0.7901
G-EIMTNet	0.8367	0.8022

As shown in **Table 6**, integrating emotion recognition and gender recognition as auxiliary tasks in depression recognition yields consistent performance enhancements across models. The improvement of MTL-Emo proves the strong correlation between emotion states and depression. The performance improvement of MTL-Gen suggests that the learned representation effectively mitigates gender bias, thereby enhancing the discriminative power of features. Notably, compared to DEP-Indep, G-EIMTNet achieves a 6.17% increase in accuracy and a 4.41% improvement in F1-score, indicating the efficacy of embedding emotional and gender information into depression recognition. Emotional states provide auxiliary clues for gender recognition; while the introduction of gender features is equivalent to establishing a physiological baseline for depression detection, enabling the model to effectively distinguish between gender-driven natural acoustic variations and depression-induced pathological acoustic anomalies. This can improve the model’s diagnostic precision.

### 4.4 t-SNE visualization

**Figure 4:**
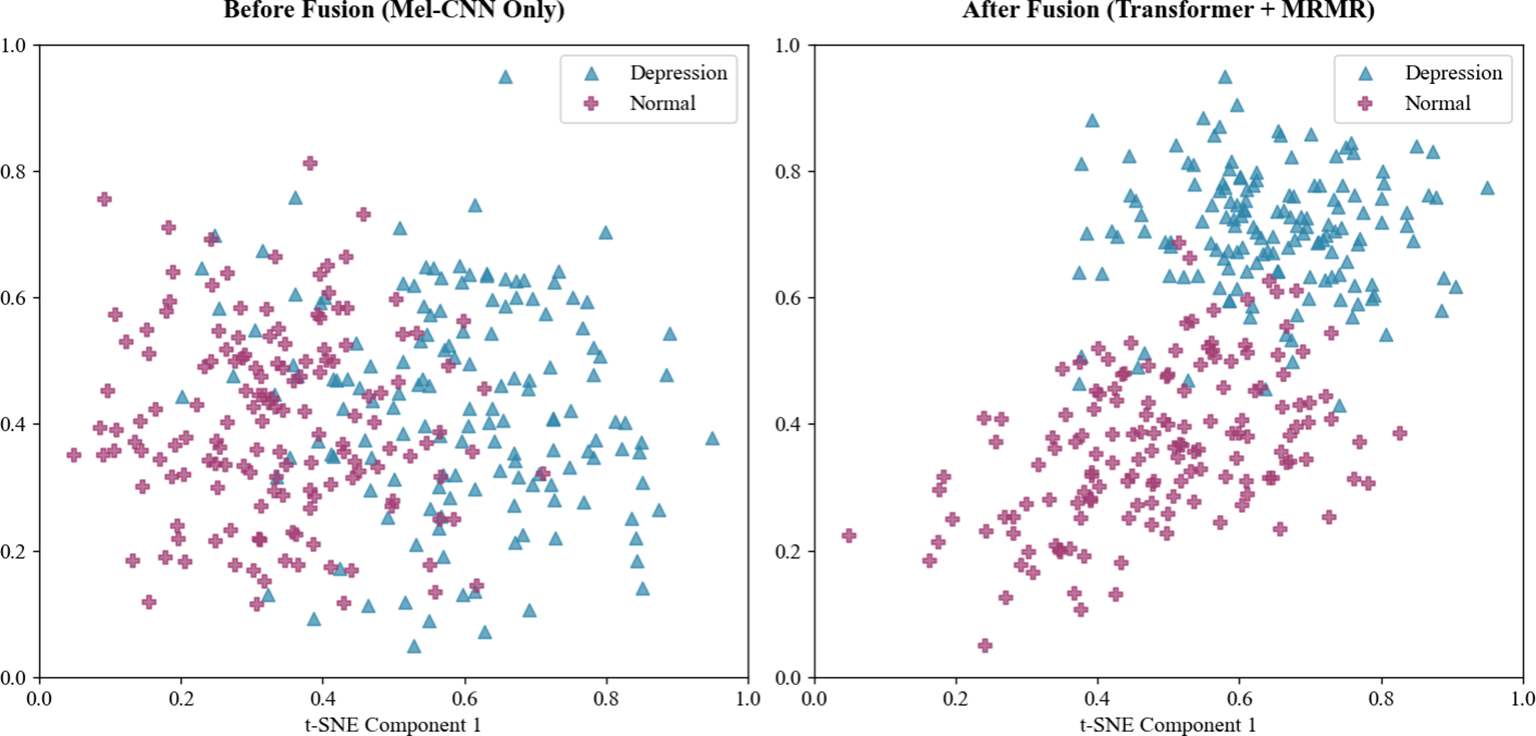
Comparison before and after transformer-based fusion.

In this experiment, t-SNE was employed to reduce the dimensionality of both Mel-CNN features and Transformer-based fusion features to a 2D plane, where scatter plots were generated to visualize and analyze the feature distribution before and after fusion. The corresponding results are presented in **Figure 4**. As can be clearly seen from **Figure 4**, after feature fusion, the depressive samples and normal samples exhibit a more distinct clustering effect, with the inter-class distance increasing and the intra-class distance decreasing. This indicates that the features fused by the Transformer demonstrate stronger discriminative power.

### 4.5 Comparison with the state-of-the-art methods

The purpose of this experiment is to conduct a comparative analysis of the performance of other methods and our proposed method. Our main comparative methods included baseline method proposed by Valstar (40), the CNN fused with LSTM for depression recognition based on Mel-Spectrogram proposed by Ma (44), the emotion-assisted multi-task CNN proposed by Dumpala (45), the gender-assisted multi-task learning proposed by Liu (46), the deep LSTM based on MFCC proposed by Rejaibi (47), spatio-temporal attention (STA) network and eigen evolution pooling(EEP) feature fusion (MAFF) strategy proposed by Niu (24), attention-based acoustic feature fusion network (ABAFnet) proposed by Xu (21), multidimensional convolutional neural networks (MDCF-Net) that incorporate emotional features proposed by Ren (48), and joint networks with tailored Attention (JTA) based on Spectrogram-Based model with Tailored Attention (STA), pre-trained model (WavLM) and Transactive Attention-based feature Fusion module (TAF) proposed by Li (23). The experimental results are showed in **Table 6**.

As indicated in **Table 7**, the early DepAudioNet model was constrained by its reliance on a single feature modality, resulting in relatively limited performance. In recent years, Dumpala and Liu have made progress by incorporating emotional and gender information into the depression recognition. Compared to DepAudioNet, their proposed methods achieving 4.18% and 3.49% improvements in accuracy respectively. This validated the predictive value of emotional and gender attributes for depression recognition. With the development of deep learning and attention mechanisms, Rejaibi employed deep LSTM for depression detection, which demonstrated the importance of capturing long-distance temporal dependencies. Niu further introduced STA and EEP, which can capture the dynamic evolution of features over time, achieving an accuracy of 0.7881 and an F1-score of 0.7681. Although Niu and Rejaibi’s method has achieved some success in capturing the temporal dynamics of features, its accuracy and F1-score were both lower than G-EIMTNet. The G-EIMTNet we proposed not only used Transformer multimodal attention to capture the temporal relationship of features and the correlation between deep features and handcrafted features (HSFs), but also incorporates emotion and gender into depression recognition. Compared with Niu’s method, the accuracy of G-EIMTNet had increased by 4.86%.

**Table 7:** Results of our method and comparative methods.

Author	Methods	Year	Accuracy	F1-score
Valstar et al. (40)	HSFs+SVM (baseline method)	2014	0.6779	0.6459
Ma et al. (44)	DepAudioNet	2016	0.7232	0.6832
Dumpala et al. (45)	Spectrogram + Emotion Task	2021	0.7650	0.7355
Liu et al. (46)	MFCC + Gender Task + Attention	2021	0.7581	0.7208
Rejaibi et al. (47)	MFCC+LSTM	2022	0.7325	0.7150
Niu et al. (24)	STA+EEP	2023	0.7881	0.7681
Xu et al. (21)	ABAFnet	2024	0.8032	0.7853
Ren et al. (48)	MDCF-Net	2025	0.7754	0.7547
Li et al. (23)	JTA(WavLM+Tailored Attention+ Transactive Attention-based feature Fusion)	2025	0.8205	0.7895
G-EIMTNet		-	0.8367	0.8022

Both the MDCF-Net proposed by Ren and the ABAFnet proposed by Xu were dedicated to integrating more features such as spectrograms, envelope features, and HSFs. However, ABAFnet implements feature fusion via dynamic weight adjustment, whereas G-EIMTNet employs Transformer-based nonlinear interactive fusion. More importantly, G-EIMTNet introduced a Gender-Emotion Interaction module. In AVEC 2014 dataset, which contain different genders and rich emotional states, simply piling up acoustic features was prone to interference from gender differences.

Compared with the JTA framework proposed by Li, the accuracy of G-EIMTNet was 1.62% higher. The JTA framework leveraged the pre-trained WavLM, whereas the G-EIMTNet achieved an accuracy of 83.67% without relying on large-scale external pre-training data. This was also credited to our fusion of deep features and handcrafted features, as well as the dedicated Gender-Emotion Interaction module proposed in this work.

**Figure 5:**
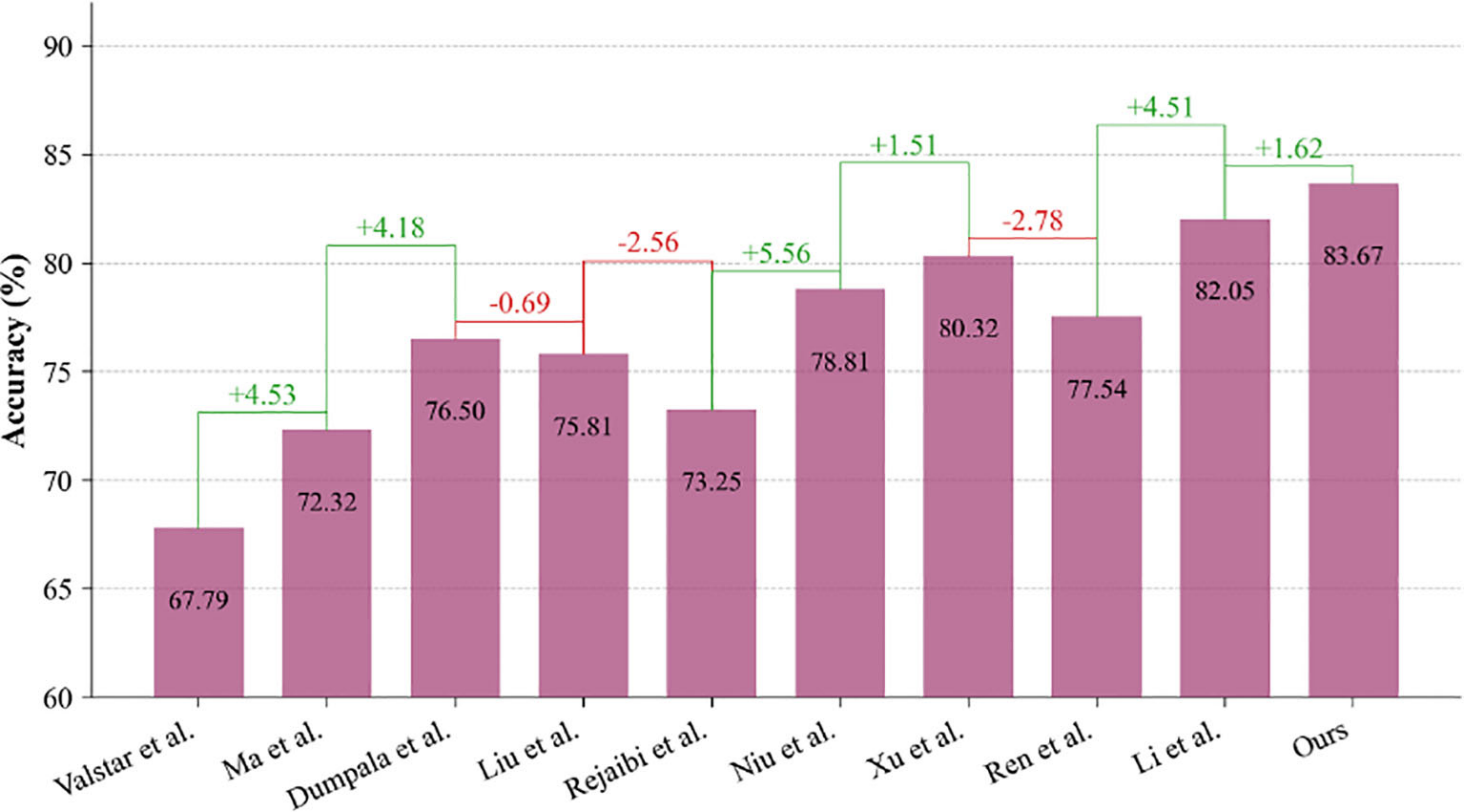
The visualization of accuracy comparison.

**Figure 6:**
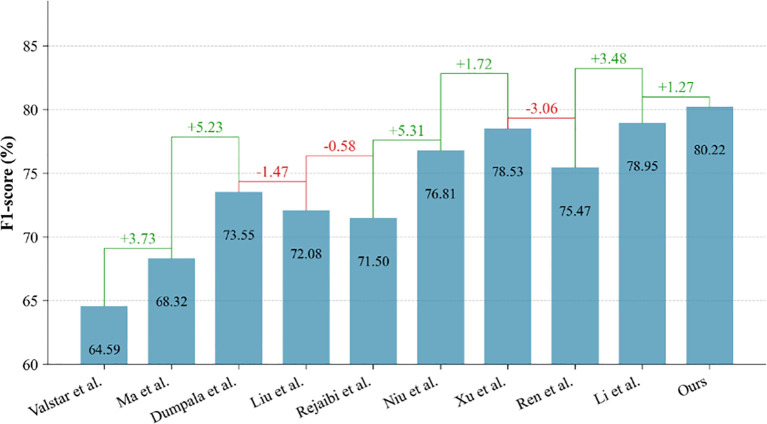
The visualization of F1-score comparison.

In conclusion, comparative analyses with state-of-the-art methods demonstrated the effectiveness of the G-EIMTNet proposed in this paper. It also indicates that depression recognition cannot be separated from emotional states, and gender differences can affect the judgment. The visualization of performance comparison is shown in **Figures 5** and **6**.

### 4.6 Discussion

The time complexity of G-EIMTNet is mainly composed of three parts: the feature extraction layer, the Transformer fusion layer, and the multi-task classifier. Among them, the time complexity of the feature extraction layer mainly depends on the convolution operation of CNN on the Mel spectrogram, which is related to the size of the convolution kernel, the number of channels, and the input image. In the Transformer fusion module, the time complexity is $O(L^{2}\cdot d)$, where *L*is the sequence length and *d* is the embedding dimension. Since speech segments are usually divided into short frames, the sequence length *L* will not be too large. The time complexity of the multi-task classifier is $O(N_{fc})$, The three parallel branches mainly consist of fully connected layers and simple convolutional pooling. The feature interaction between tasks only involves vector operations, with extremely low complexity. Therefore, compared to training three separate models for each task, G-EIMTNet significantly reduces the computing time through the parameter sharing mechanism.

## 5 Conclusions

This work presents the Gender-Emotion Interaction Multi-task Network (G-EIMTNet), a novel framework designed to enhance the robustness of speech-based depression recognition. Our method targets two critical limitations in existing research: first, the lack of effective fusion strategies for heterogeneous acoustic features; second, the oversight of intrinsic interactions among depression, emotion, and gender. In our method, deep Mel-spectral representations from CNN are first fused with MRMR-selected HSFs via a Transformer-based multimodal attention mechanism, which bridges their semantic gap and enables adaptive alignment and enhancement. We further innovatively construct a multi-task interactive framework: unlike traditional methods treating gender or emotion as isolated labels, G-EIMTNet explicitly models their coupling mechanism, with experimental results on the AVEC 2014 dataset validating the method’s effectiveness.

Despite the promising results, this work still has limitations that warrant further improvement. G-EIMTNet introduces multiple loss weights, $\lambda_{1}$ and $\lambda_{2}$. Finding the optimal weights requires a large number of grid search experiments, and the parameter tuning cost is relatively high. Furthermore, although the transformer excels at local feature fusion, for a disorder like depression, which exhibits long-term behavioral patterns, the model currently tends to analyze short-time speech segments, possibly neglecting more macroscopic trends in intonation changes or long-term psychological fluctuations. Therefore, in subsequent work, we will explore an adaptive weighting strategy for the joint loss function in multi-task learning and simultaneously consider integrating long-term behavioral features into depression recognition to further improve performance.

## Data Availability

Publicly available datasets were analyzed in this study. This data can be found here: http://avec2013-db.sspnet.eu/.
